# Elevation in network dynamics amplifies amyloid‐dependent tau pathology

**DOI:** 10.1002/alz.71354

**Published:** 2026-04-14

**Authors:** Jieying Li, Yang Yi, Lin Gan, Gleb Bezgin, Tevy Chan, Nesrine Rahmouni, Yi‐Ting Wang, Etienne Aumont, Seyyed Ali Hosseini, Brandon J. Hall, Lydia Trudel, Joseph Therriault, Arthur C. Macedo, Kely Monica Quispialaya Socualaya, Jaime Fernandez Arias, Yansheng Zheng, Delphine Olivia‐Lopez, Robert Hopewell, Chris Hung‐Hsin Hsiao, Ting Zou, Jean‐Paul Soucy, Serge Gauthier, Paolo Vitali, Tharick A. Pascoal, Qolamreza R. Razlighi, Maxime Montembeault, Rong Li, Pedro Rosa‐Neto

**Affiliations:** ^1^ Translational Neuroimaging Laboratory Department of Neurology and Neurosurgery McConnell Brain Imaging Centre Montreal Neurological Institute McGill University Montreal Quebec Canada; ^2^ Sichuan Provincial Centre for Mental Health Sichuan Provincial People's Hospital, School of Medicine University of Electronic Science and Technology of China Chengdu China; ^3^ McGill University Research Centre for Studies in Aging Douglas Hospital Research Centre Montreal Québec Canada; ^4^ The Clinical Hospital of Chengdu Brain Science Institute Brain‐Computer Interface & Brain‐Inspired Intelligence Key Laboratory of Sichuan Province School of Life Science and Technology University of Electronic Science and Technology of China Chengdu China; ^5^ Douglas Research Centre & Department of Psychiatry McGill University Montréal Quebec Canada; ^6^ PET Unit, McConnell Brain Imaging Centre Montreal Quebec Canada; ^7^ Department of Psychiatry School of Medicine University of Pittsburgh Pittsburgh Pennsylvania USA; ^8^ Department of Radiology Brain Health Imaging Institute Weill Cornell Medicine New York New York USA; ^9^ Peter O'Donnell Jr. Brain Institute (OBI) University of Texas Southwestern Medical Centre (UTSW) Dallas Texas USA

**Keywords:** Alzheimer's disease, Braak staging, brain network, cognition, pathology

## Abstract

**INTRODUCTION:**

The role of brain network dynamics in relation to amyloid beta (Aβ) and tau pathology across Braak stages remains unclear.

**METHODS:**

In this cross‐sectional study of 216 participants from Translational Biomarkers of Aging and Dementia (TRIAD) cohort, we analyzed resting‐state functional magnetic resonance imaging using a multilayer modularity algorithm to assess brain network dynamics across 10 predefined functional networks, stratified by amyloid and tau positron emission tomography biomarkers and Braak stages.

**RESULTS:**

Switching rates were significantly elevated in Aβ‐positive/tau‐positive individuals relative to Aβ‐negative/tau‐negative individuals, and increased progressively with advancing Braak stages. Elevated switching rates were strongly correlated with Aβ and tau burden in dorsal attention network and sensorimotor network, as well as with cognitive severity. Importantly, the interaction between network switching rate and Aβ burden synergistically contributed to accelerated tau accumulation in Braak stage III to V regions.

**DISCUSSION:**

These findings support the framework that increased network switching may amplify Aβ‐related tau load and cognitive deterioration in Alzheimer's disease.

## Highlights

- Amyloid beta (Aβ)‐positive/tau‐positive individuals showed significantly higher switching rates compared to Aβ‐negative/tau‐negative individuals, across global network as well as the dorsal attention network (DAN), default mode network, frontoparietal network, salience network, sensorimotor network (SMN), and visual network.
- Switching rates increased progressively across all participants in accordance with advancing tau positron emission tomography Braak stages.
- Elevated network switching rates were significantly associated with global Aβ and tau burden in the DAN and SMN, and showed strong correlations with cognitive impairment.
- A significant interaction was observed between network switching rate and Aβ burden, which synergistically facilitated the accelerated accumulation of tau pathology in Braak stage III to V regions.

## 1 BACKGROUND

Alzheimer's disease (AD) represents the most common cause of dementia. It is characterized by the accumulation of amyloid beta (Aβ) plaques, tau neurofibrillary tangles, and progressive cognitive impairment.^1^ Neuroimaging biomarkers, such as amyloid positron emission tomography (PET) and tau PET imaging, have emerged as powerful tools for detecting AD pathology, even prior to the onset of overt clinical symptoms.^2^ Tau accumulation contributes to neurodegeneration and progressive cognitive decline^3^ and exhibits a strong association with AD progression. This accumulation is quantified by the Braak staging system, beginning in the transentorhinal cortex (stage I), advancing to the entorhinal cortex and hippocampus (stage II), inferior temporal neocortex (stage III), association cortices (stages IV and V), and ultimately reaching the primary sensory and motor cortices (stage VI).^4^, ^5^ PET‐based Braak stages are stage‐specific markers that indicate the magnitude of pathological tau hyperphosphorylation. They have been conceptualized to show associations with the severity of dementia,^6^ making them particularly valuable for tracking AD progression.^7^, ^8^

Although Aβ is thought to initiate the accumulation of tau from transentorhinal and entorhinal regions to the neocortex,^9^ the spatial distribution and progression pattern of tau accumulation diverge substantially from those of widespread neocortical Aβ accumulation, with tau pathology following a more hierarchical and regionally selective trajectory.^10^ Growing evidence suggests that the intrinsic network architecture of the human brain may underlie the distinct regional distribution of tau pathology in AD;^11^, ^12^, ^13^ however, how brain network dynamic and connectivity contribute to tau propagation and disease severity in vivo remains unclear and requires further investigation.

Multilayer brain network dynamics capture the flexibility and stability of brain functional networks. In the normal brain, in a given time frame, one observes dynamic connectivity switching across brain regions. This metric offers insights into how the brain dynamically reconfigures over time, frequency, and modality.^14^, ^15^ Dynamic connectivity studies reveal that abnormal or unstable switching is associated with various brain conditions.

Here, we investigate these associations with tau propagation and AD severity using the network switching rate, also known as flexibility. This approach offers a more temporally sensitive view of brain network reorganization than static functional connectivity, which may fail to detect subtle disruptions in early disease stages.^16^ By capturing moment‐to‐moment changes in network configuration, network switching rate overcomes the limitations of static functional connectivity and may more accurately reflect the dynamic alterations associated with AD pathology.

In this cross‐sectional study, we first aimed to identify significant differences in network switching rate across the AD continuum and Braak staging groups. We then investigated the associations between network switching rate, amyloid burden, tau pathology progression, and clinical severity, with particular focus on Braak stage–specific patterns. Finally, we examined whether network switching rate modulated the effect of amyloid burden on tau propagation and cognitive decline, particularly within regions corresponding to higher Braak stages. We hypothesized that elevated multilayer network switching rate amplifies the pathological effects of amyloid burden, facilitating greater tau deposition across Braak stage regions in AD.

## 2 METHODS

### 2.1 Participants

We assessed 216 participants enrolled in the Translational Biomarkers of Aging and Dementia (TRIAD) cohort for whom resting‐state functional magnetic resonance imaging (rs‐fMRI) data were acquired and passed the manual quality check assurance before and after image preprocessing. All participants also underwent Aβ PET imaging with [^18^F]AZD4694 and tau PET imaging with [^18^F]MK6240. Participants underwent clinical and cognitive assessment, including the Clinical Dementia Rating (CDR) score, the Mini‐Mental State Examination (MMSE), and neuropsychological testing. Cognitively normal (CN) individuals had no objective cognitive impairment, MMSE score ≥ 24, CDR global = 0, and were classified as not depressed. Aβ‐negative participants with a diagnosis other than CN were excluded owing to suspected non‐AD pathology. Each of the 216 participants was classified as Aβ positive or negative (A+/−) based on established global [^18^F]‐AZD4694 (global standardized uptake value ratio [SUVR] > 1.55),^17^ and tau positivity (T+/−) was based on [^18^F]‐MK6240 (meta region of interest [ROI] SUVR > 1.30),^18^ resulting in 68 A+T+ participants, 47 A+T− participants, and 101 A−T− participants.

The study was approved by the Montreal Neurological Institute (MNI) PET working committee and the Douglas Mental Health University Institute Research Ethics Board. Written informed consent was obtained for all participants.

**RESEARCH IN CONTEXT**

1. **Systematic review**: The authors reviewed the literature (PubMed and Google Scholar) using traditional sources. Several studies have reported altered brain network dynamics in aging and Alzheimer's disease, However, few have systematically investigated switching rate in relation to both amyloid beta (Aβ) and tau accumulation, or mapped these changes across tau positron emission tomography Braak staging in large multimodal datasets.
2. **Interpretation**: Our findings indicate that switching rates are progressively elevated across advancing tau Braak stages and are significantly higher in Amyloid beta (Aβ)‐positive/tau‐positive individuals relative to Aβ‐negative/tau‐negative individuals. Elevated switching rates were also strongly associated with both Aβ and tau burden in the dorsal attention network and sensorimotor network, as well as with worsening cognitive performance. Moreover, we observed a synergistic interaction between network switching rate and Aβ burden. These findings support the framework that increased network switching amplifies Aβ‐related tau spread and cognitive deterioration.
3. **Future directions**: Future research should explore whether switching rate can predict longitudinal tau propagation, cognitive decline, or response to therapeutic interventions. Additionally, mechanistic studies are needed to determine how Aβ burden and network flexibility interact to influence tau spread across distinct functional circuits.

### 2.2 MRI and PET acquisition

MRI was acquired at the MNI using a 3‐Tesla Siemens Magnetom Prisma scanner. rs‐fMRI was acquired using a multiband echo planar imaging sequence (repetition time [TR]) = 681 ms; echo time [TE] = 32 ms; flip angle = 90°; scanning matrix = 88 × 88; field of view = 220 × 220 mm^2^; voxel size = 2.5 × 2.5 × 2.5 mm^3^; 54 slices). Each sequence lasted 9.9 minutes, resulting in 870 volumes. T1‐weighted images were also acquired for each subject with a magnetization‐prepared rapid gradient echo sequence (MPRAGE; TR = 2300 ms; TE = 2.96 ms; flip angle = 90°; voxel size = 1.0×1.0×1.0 mm^3^).

PET data were acquired using a Siemens High‐Resolution Research Tomograph. For [^18^F]MK6240 PET, images were acquired between 90 to 110 minutes after an intravenous bolus injection of the radiotracer.^19^ [^18^F]AZD4694 PET images were acquired between 40 to 70 minutes after the intravenous bolus injection of the radiotracer.^17^ Attenuation correction for each PET session was performed using a 6‐minute transmission scan using a rotating 137Cs point source, conducted immediately after the PET imaging. Additional information can be found in our previous publication.^17^, ^19^

### 2.3 MRI and PET preprocessing

Pre‐processing of the rs‐fMRI data was carried out using the DPARSF software (http://www.rfmri.org/DPARSF).^20^ The first 10 volumes were discarded to allow for steady‐state magnetization effects. The common step of slice‐timing correction was omitted due to the short TRs, to avoid the potential spreading effect of artifacts or outlier scans.^21^ Spatial realignment to the first volume was performed to correct for motion artifacts. To denoise the images, nuisance covariates, including white matter and cerebrospinal fluid signals^22^ as well as a 24‐parameter head motion^22^ were regressed out, followed by band‐pass filtering (0.01–0.1 Hz) provided in the native space. Participants who demonstrated excessive head movement (> 3 mm translation, > 3 degrees rotation, and mean framewise displacement [FD] Jenkinson > 0.2)^23^ were excluded from further analysis. This resulted in the exclusion of 13 A+T+ participants, 4 A+T− participants, and 16 A−T− participants. rs‐fMRI was then transformed to the MNI template space and spatially smoothed with a 6 mm full width at half‐maximum (FWHM) Gaussian kernel.

PET images were automatically registered to corresponding T1‐weighted anatomical images, which were subsequently spatially normalized to the MNI template using linear and non‐linear transformations. [^18^F]MK6240 PET images underwent meninges stripping in native space to minimize spillover effects prior to normalization and smoothing. SUVR calculations used inferior cerebellar gray as the reference for [^18^F]MK6240^14^ and whole cerebellar gray matter for [^18^F]AZD4694. ^17^ PET images were spatially smoothed to an 8‐mm FWHM resolution. The global [^18^F]AZD4694 SUVR composite encompassed precuneus, prefrontal, orbitofrontal, parietal, temporal, and cingulate cortices, with positivity defined by an SUVR > 1.55.^17^ The temporal meta‐ROI [^18^F]MK6240 SUVR composite encompassed the entorhinal, parahippocampal, amygdala, fusiform, inferior, and middle temporal cortices, with positivity defined as SUVRs > 1.30.^18^ Participants were also categorized in PET‐based Braak stages according to the topography of tau PET abnormality as described previously.^7^

### 2.4 Dynamic functional network construction

For each participant, a dynamic functional network was generated using a sliding‐window approach. rs‐fMRI time series were extracted from 264 ROIs defined by the Power 264 functional atlas,^24^ encompassing 10 predefined functional networks: the auditory network (AN), cingulo‐opercular network (CO), dorsal attention network (DAN), default mode network (DMN), frontoparietal network (FPN), salience network (SAN), sensorimotor network (SMN), subcortical network (SUB), ventral attention network (VAN), and visual network (VN). Prior research suggested that a 30‐ to 60‐second time window effectively captures fluctuations in resting‐state dynamic functional connectivity (FC), balancing specificity (a sufficiently long window length for reliably estimate dynamic FC fluctuations) with sensitivity (sufficiently short window length to avoid overlooking actual dynamic FC changes).^25^, ^26^ In this study, a sliding‐window length of 74 TRs (≈ 50.4 seconds) with a step size of 3 TRs was used, a methodology that has been previously documented in the prior literature.^27^, ^28^, ^29^, ^30^ To ensure that the primary findings reported here were not confounded by the key parameter of window length, additional analyses were performed using alternative window lengths (≈ 40 seconds and ≈ 60 seconds). These validation analyses yielded comparable results (see Figure S1 and S2 in supporting information). Within each time window, Pearson correlation coefficients were computed between all ROI pairs to construct time‐resolved FC matrices, which were subsequently Fisher *z* transformed to normalize the correlation values. To characterize dynamic changes in network organization over time, we applied a Louvain‐like multilayer community detection algorithm.^31^ This algorithm assigns each ROI to a community (i.e., a module) in each time window, with the goal of maximizing modularity across temporal layers. ROIs that frequently switch community assignments across time windows are considered dynamically flexible. All parameters used in the community detection procedure were consistent with previous work,^32^ and modularity optimization was repeated 100 times per participant to ensure solution robustness.^33^

### 2.5 Network switching rate

Switching rate (also referred to as flexibility) quantifies the temporal variability of brain network organization by measuring how frequently a given ROI changes its community assignment across consecutive time windows.^34^ A higher switching rate reflects increased dynamic reconfiguration and reduced temporal stability of network affiliation. For each participant, switching rates were calculated for all 264 ROIs. ROIs were then grouped into their corresponding functional networks based on the Power atlas labels. The network‐specific switching rate was defined as the mean switching rate across all ROIs belonging to a given functional network. This metric reflects the average temporal flexibility of each large‐scale functional system. The global switching rate was computed as the mean switching rate across all ROIs, representing overall brain network dynamics.

### 2.6 Statistical analysis

All statistical analyses were performed in R (version 4.4.2). Group differences in demographic, cognitive, genetic, and PET variables were assessed using analysis of variance (ANOVA) for normally distributed continuous variables, the Kruskal–Wallis test for non‐normally distributed variables, and chi‐squared (*χ* ^2^) tests for categorical data (Table 1).

**TABLE 1:** Demographics and clinical characteristics of theTRIAD cohort.

Variables	A+T+(*n* = 68)	A+T−(*n* = 47)	A–T−(*n* = 101)	*p* value
Sex (female/male)	39/29	29/18	65/36	0.6561
Age (years)	68.7 (8.43)	71.3 (10.4)	70.3 (5.58)	0.177
Education	15.1 (3.71)	15.6 (4.29)	15.7 (3.38)	0.554
MMSE	24.7 (5.16)	28.6 (2.38)	29.2 (0.889)	< 0.001
MoCA	19.4 (7.19)	27.0 (2.11)	28.1 (1.85)	< 0.001
CDR‐Global	0.7 (0.48)	0.2 (0.28)	0 (0)	< 0.001
RAVLT‐Delayed	3.65 (4.58)	9.10 (3.64)	10.9 (2.92)	< 0.001
*APOE* ε4	69.1%	27.7%	20.8%	< 0.001
Global Aβ SUVR	2.44(0.41)	2.02(0.38)	1.30(0.09)	< 0.001
Meta‐ROI tau SUVR	2.40(1.02)	1.10(0.10)	1.04(0.10)	< 0.001

*Note*: Means ± SDs are displayed for continuous measures.

Abbreviations: A, amyloid beta; Aβ, amyloid beta; *APOE*, apolipoprotein E; CDR‐Global, Clinical Dementia Rating Global score; Meta‐ROI, medial temporal region of interest; MMSE, Mini‐Mental State Examination; MoCA, Montreal Cognitive Assessment; RAVLT‐Delayed, Rey Auditory Verbal Learning Test—Delayed Recall; SUVR, standardized uptake value ratio; T, tau; TRIAD, Translational Biomarkers of Aging and Dementia.

To investigate whether switching rates were elevated across the AD continuum, we compared global and network‐specific switching rates across three biomarker‐defined groups: A+T+, A+T−, and A−T−. One‐way ANOVA was used to test for group differences, followed by least square difference (LSD) post hoc comparisons (significance threshold: *P* ≤ 0.05, performing false discovery rate [FDR] correction for multiple testing). Participants were also stratified by PET‐based Braak stage into four categories: Stage 0, I to II, III to IV, and V to VI. Group differences in switching rates across Braak stages were assessed using one‐way ANOVA, a Welch ANOVA, or the Kruskal–Wallis test, depending on normality and variance homogeneity. Post hoc pairwise comparisons were performed using the LSD method, without correction for multiple testing.

Voxel‐wise linear regressions were conducted using the mincLm package in RMINC to examine associations between network switching rate and Aβ or tau PET SUVRs, controlling for age and sex. Multiple comparisons were corrected using random field theory, with a cluster‐level threshold of *P* < 0.001.^35^ To test the hypothesis that switching rate reflects tau progression, we performed Spearman correlation analyses between switching rate and tau PET SUVR across Braak stage I to VI ROIs, adjusting for age and sex. Linear regression models were used to assess the relationship between rescaled switching rates and *z* scored cognitive performance measures (MMSE, Montreal Cognitive Assessment [MoCA], Rey Auditory Verbal Learning Test—Delayed Recall [RAVLT‐Delayed], and CDR Sum of Boxes [CDR‐SOB]), controlling for age, sex, and education. For each functional network, β coefficients and associated *P* values were extracted.

To evaluate whether switching rate modulates the effect of amyloid on tau accumulation and cognitive decline, we tested interaction models with global Aβ SUVR × switching rate as predictors of tau PET SUVR across Braak ROIs and cognitive scores (MMSE, MoCA, RAVLT‐Delayed, and CDR‐SOB). Covariates included age, sex, Aβ SUVR, and switching rate. Interaction robustness was assessed via 1000 iterations bootstrapped regressions to derive 95% confidence intervals. Model‐based simulations were used to estimate predicted tau values across switching rate gradients, visualized across amyloid burden bins. All *P* values were corrected for multiple comparisons using the FDR method.

## 3 RESULTS

There were no significant differences in sex, age, or years of education between the A+T+, A+T–, and A−T− groups. The A+T+ group showed significantly lower MMSE, MoCA, and RAVLT‐Delayed scores; higher CDR scores; and a higher proportion of apolipoprotein E ε4 carriers compared to the other groups. Demographics and clinical characteristics, as well as Aβ and tau PET SUVR values are presented in Table 1.

### 3.1 Elevated global and network switching rate in A+T+ group

**FIGURE 1:**
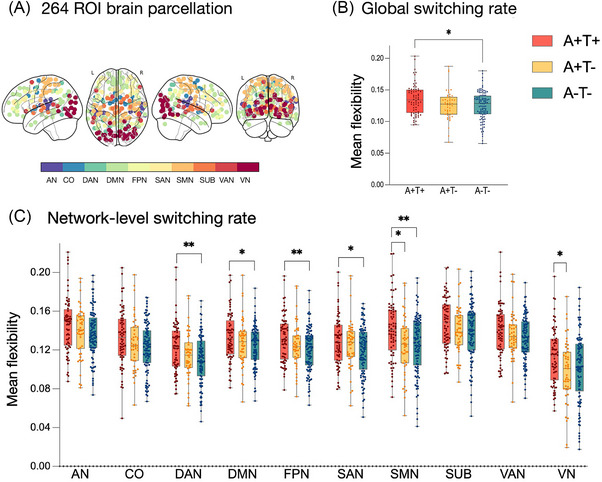
Tau positivity imposes a variable reduction on flexibility across brain networks. Summary of network switching rate analysis across groups. A, Brain parcellation based on the Power 264 functional atlas, comprising 264 ROIs assigned to 10 canonical networks. B, Global switching rate was significantly higher in the A+T+ group compared to the A−T− group (*P *< 0.05 FDR corrected). C, Significant group differences in network‐specific switching rates were observed in DAN, DMN, FPN, SAN, SMN, and VN (*p* < 0.05). The A+T+ group showed elevated switching rates compared to A−T− (DAN, DMN, FPN, SAN, SMN) and A+T− (SMN, VN). Error bars indicate standard deviation. *p < 0.05; **P < 0.01. A, amyloid beta; AN, auditory network; CO, cingulo‐opercular network; DAN, dorsal attention network; DMN, default mode network; FDR, false discovery rate; FPN, frontoparietal network; ROI, region of interest; SAN, salience network; SMN, sensorimotor network; SUB, subcortical network; T, tau; VAN, ventral attention network; VN, visual network.

We first examined group differences in global network switching rate across the three groups. We found a significant increase in whole‐brain switching rate in the A+T+ group (*F* = 4.421, *p* = 0.013); post hoc analysis indicated that the A+T+ group had significantly higher switching rates than the A−T− group (FDR‐corrected *p* = 0.017; Figure 1B).

Next, we assessed switching rates within each of the 10 networks defined by the Power 264 functional atlas (Figure 1A). Significant group effects were observed in the DAN (*F* = 4.492, *p* = 0.012), DMN (*F* = 3.223, *p* = 0.042), FPN (*F* = 4.672, *p* = 0.010), SAN (*F* = 3.270, *p* = 0.040), SMN (*F* = 6.559, *p* = 0.002), and VN (*F* = 3.554, *p* = 0.030). Post hoc comparisons showed that the A+T+ group exhibited significantly elevated switching rates compared to the A−T− group in the DAN, DMN, FPN, SAN, and SMN (FDR‐corrected *p* = 0.017, 0.040, 0.017, 0.040, 0.017, respectively). Additionally, switching rates in the SMN and VN were significantly higher in the A+T+ group than in the A+T− group (FDR‐corrected *p* = 0.017, 0.040; Figure 1C).

### 3.2 Network switching rate increases with advancing Braak stage

**FIGURE 2:**
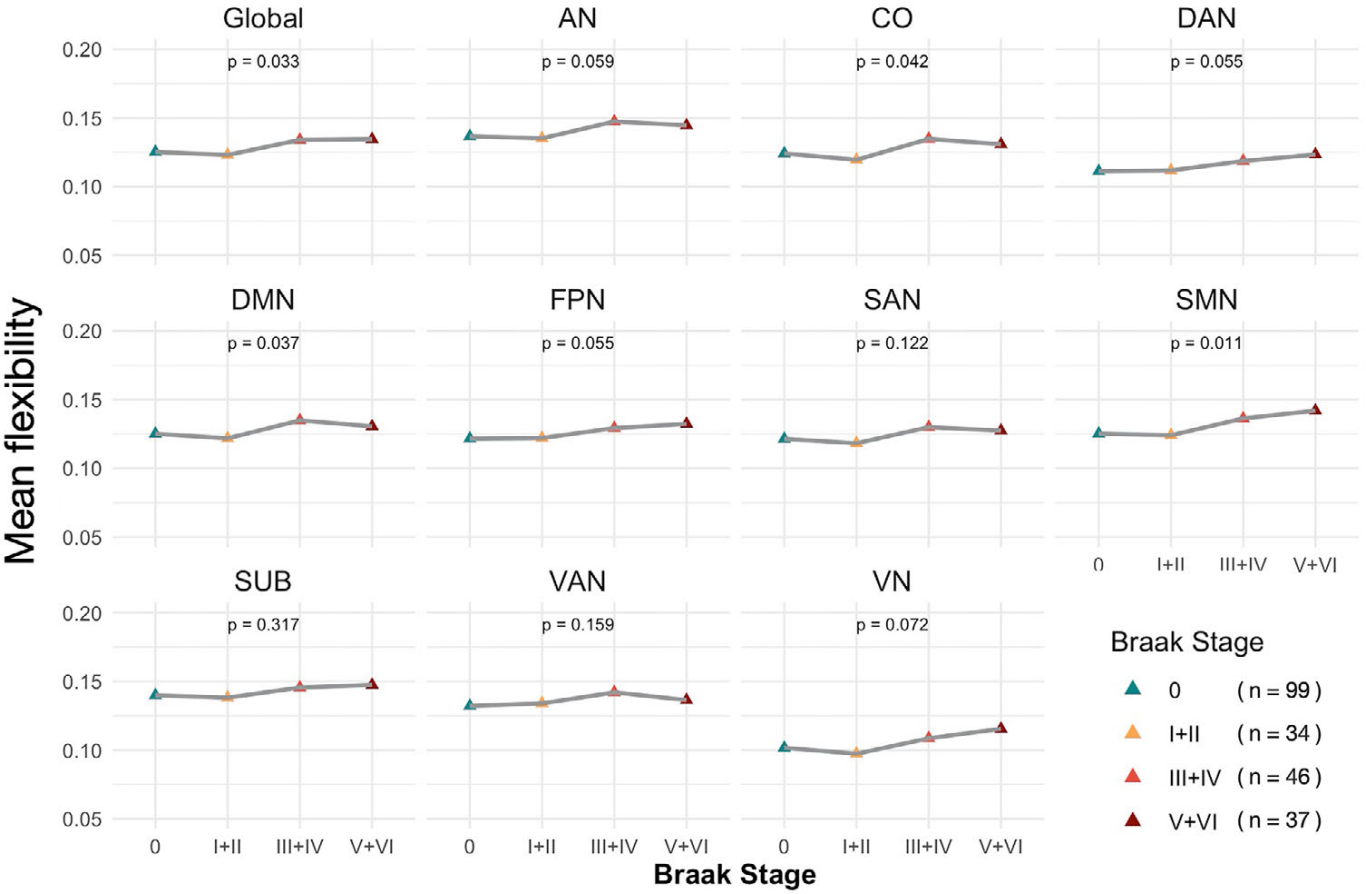
Network switching rate across PET‐based Braak stages. Participants were grouped into four categories based on tau PET Braak staging: Stage 0 (no abnormality), Stage I to II (transentorhinal involvement), Stage III to IV (limbic spread), and Stage V to VI (isocortical involvement). Triangle represent the mean value of switching rate. AN, auditory network; CO, cingulo‐opercular network; DAN, dorsal attention network; DMN, default mode network; FPN, frontoparietal network; PET, positron emission tomography; SAN, salience network; SMN, sensorimotor network; SUB, subcortical network; VAN, ventral attention network; VN, visual network.

We examined the differences in switching rate across four groups categorized by PET‐based Braak staging: Stage 0 (no tau pathology), Stage I to II (transentorhinal), Stage III to IV (limbic), and Stage V to VI (isocortical involvement). A progressive increase in switching rate was observed across stages. We found significant differences in the global network as well as in the CO, DMN, and SMN (FDR corrected; Figure 2). However, no pairwise differences between groups remained significant after FDR correction (*p* > 0.05 for all post hoc comparisons).

### 3.3 Network switching rate negatively correlates with cognitive score

**FIGURE 3:**
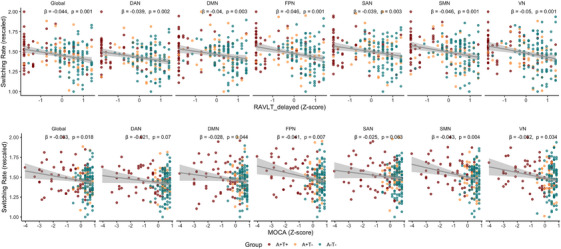
Increased network switching rate is associated with low cognitive scores. Regression lines reflect group‐independent associations between cognitive performance (RAVLT‐Delayed or MoCA) and switching rates, adjusted for age, sex, and education. Annotated β coefficients and FDR‐adjusted *p* values indicate the strength and significance of these associations. AN, auditory network; CO, cingulo‐opercular network; DAN, dorsal attention network; DMN, default mode network; FDR, false discovery rate; FPN, frontoparietal network; MoCA, Montreal Cognitive Assessment; RAVLT‐Delayed, Rey Auditory Verbal Learning Test—Delayed Recall; SAN, salience network; SMN, sensorimotor network; SUB, subcortical network; T, tau; VAN, ventral attention network; VN, visual network.

We next examined the associations between network switching rates and cognitive performance, evaluated using the MMSE, MoCA, RAVLT‐Delayed, and CDR‐SOB scores. Switching rate was positively correlated with RAVLT‐Delayed in the global network and in several functional networks, including the DAN, DMN, FPN, SAN, SMN, and VN (Figure 3). Additionally, significant positive associations were observed between switching rate and MoCA scores in the global network, as well as in the DMN, FPN, SMN, and VN networks (Figure 3). In contrast, no significant correlations were observed between switching rate and MMSE or CDR‐SOB scores. Lower RAVLT‐Delayed and MoCA scores were generally associated with increased switching rates, suggesting a negative link between cognitive performance and dynamic network flexibility in AD spectrum. Then, we tested whether network switching rate and amyloid burden have interaction effects on cognitive performance. However, we observed no significant interaction between global amyloid burden and network‐specific switching rates in relation to cognitive scores.

### 3.4 Network switching rate positively correlates with amyloid and tau

**FIGURE 4:**
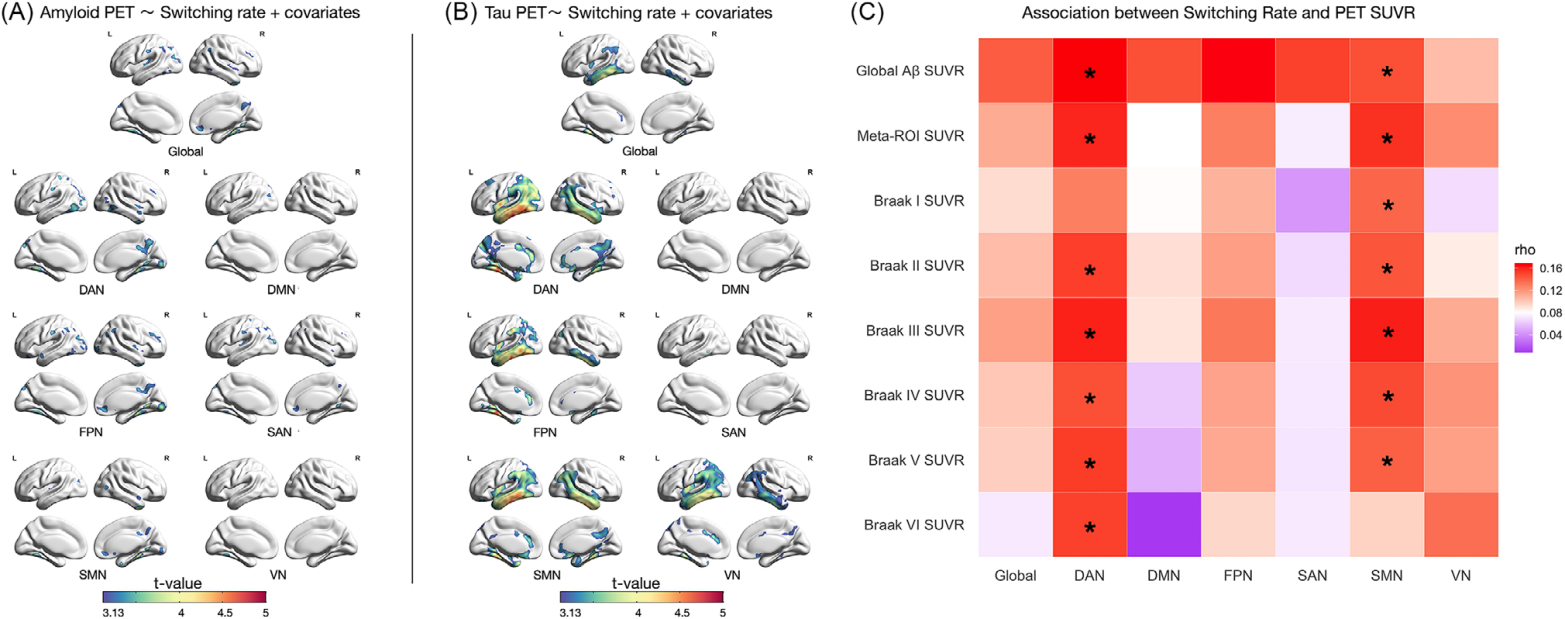
Tau imposes a regional higher network switching rate in brain pathways associated with tau propagation. T‐statistical parametric maps show the voxelwise associations between global and network‐specific switching rates and PET for [^1^ ^8^F]AZD4694 amyloid (A) and [^1^ ^8^F]MK6240 tau (B). Voxelwise analyses were conducted using linear models controlling for age and sex. Results are displayed at a voxelwise threshold of *p* < 0.001 and corrected for multiple comparisons. C, Spearman rho values for the associations between switching rates across functional networks and PET‐derived amyloid (global SUVR) and tau burden (Braak stage I−VI ROIs and meta‐ROI SUVRs). Asterisks denote levels of statistical significance after FDR correction: *p* < 0.05 (*), *p* < 0.01 (**). Aβ, amyloid beta; DAN, dorsal attention network; DMN, default mode network; FDR, false discovery rate; FPN, frontoparietal network; PET, positron emission tomography; ROI, region of interest; SAN, salience network; SMN, sensorimotor network; SUVR, standardized uptake value ratio; VAN, ventral attention network; VN, visual network.

We next examined the association between global and network‐specific switching rates and AD pathology. Voxel‐wise analyses, controlling for age and sex, revealed that global and increased switching rate networks were positively correlated with amyloid burden in the temporal, parietal, and occipital cortices (*p* < 0.001; Figure 4A). Similarly, switching rate showed significant positive correlations with tau load in the medial temporal and tempoparietal lobes, as well as in the cingulate cortex (*p* < 0.001; Figure 4B).

Spearman correlation analysis revealed significant positive associations between global amyloid PET SUVR and switching rate in the DAN (*r* = 0.17, *p* = 0.034, FDR corrected) and in the SMN (*r* = 0.15, *p* = 0.039, FDR corrected; Figure 4C). We also observed that higher switching rates were significantly correlated with tau PET meta‐ROI SUVR in the DAN (*r* = 0.16, *p* = 0.034) and in the SMN (*r* = 0.16, *p* = 0.046). Furthermore, switching rate was positively associated with Braak stage–based tau SUVRs: specifically, with Braak stage II to VI regions in the DAN and with Braak stage I to V regions in the SMN (all FDR‐corrected *p* < 0.05; Figure 4C).

### 3.5 Switching rate and amyloid burden interact to accelerate tau accumulation

**FIGURE 5:**
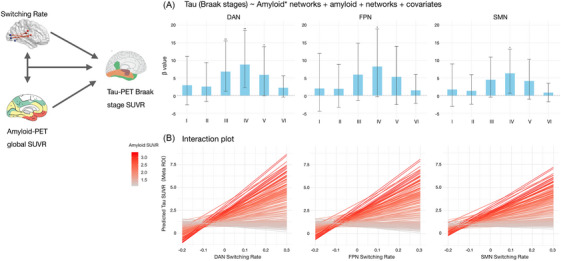
Network switching rate and amyloid burden interact to influence tau accumulation. A, Bar plot showing the interaction effects between global amyloid SUVR and DAN, FPN, SMN network switching rate on tau accumulation across Braak stages I to VI. The *y* axis reflects the interaction β estimate from linear models, with error bars representing 95% confidence intervals obtained via bootstrapping. Asterisks denote levels of statistical significance after FDR correction (*p*  <  0.05*, *p*  <  0.01**, *p*  <  0.001***). B, Line plot illustrating model‐based predictions of tau SUVR (meta‐ROI) across a range of switching rate values. Each line represents an individual participant, with predicted tau values derived from a linear model including amyloid × switching rate interaction. Line color encodes raw amyloid SUVR, highlighting that participants with higher amyloid levels (red) exhibit steeper increases in tau burden with increasing switching rates, reflecting a synergistic effect. DAN, dorsal attention network; FDR, false discovery rate; FPN, frontoparietal network; ROI, region of interest; SMN, sensorimotor network; SUVR, standardized uptake value ratio.

We tested whether network switching rate and amyloid burden have synergistic effects on accelerating tau load. We observed a significant interaction between global amyloid burden and network‐specific switching rates in predicting regional tau load. Regionally, elevated switching rates within the DAN were significantly associated with increased tau SUVR in Braak stages III to V (*p* = 0.009, 0.002, 0.02, respectively), and higher FPN and SMN switching rates significantly amplified tau SUVR in Braak stage IV (*p* = 0.018, *p* = 0.012, respectively; Figure 5A). This suggests that instability within these functional networks may play a role in facilitating tau spread during the intermediate stages of AD progression. Model‐based simulations further revealed that this interaction effect was more pronounced in individuals with high amyloid SUVR, as shown by steeper increases in predicted tau meta‐ROI across switching rate bins (Figure 5B).

## 4 DISCUSSION

In this study, we observed elevated network switching rates in the A+T+ group, but not in the A+T− group, indicating that the presence of tau pathology may play a critical role in modulating dynamic FC. Furthermore, network switching rates progressively increased with advancing tau PET Braak stages and were associated with worsening cognitive performance. Notably, we observed a synergistic interaction between global Aβ burden and network switching rate, which markedly enhanced the topographical accumulation of tau pathology. These findings support the hypothesis that progressive disruption of functional network dynamics contributes to the acceleration of AD pathology.

We also found that global and network‐specific switching rate is elevated in individuals on the AD continuum compared to CN controls and were strongly associated with cognitive severity, specifically in the DAN, DMN, FPN, SAN, and SMN. Excessive network switching rate suggests greater instability or more frequent transitions between brain states of segregated and integrated information processing.^36^ ^37^ This aligns with previous findings demonstrating that increased network switching rate is associated with reduced global network connectivity in healthy aging populations,^14^ and may represent a dysfunctional network state that compromises efficient communication across large‐scale brain systems. A prior study found older participants having higher global and network‐specific switching rate,^38^ raising questions about whether such changes are compensatory mechanisms or early signs of network instability that could lead to cognitive impairments. Notably, another study reported that switching rate is higher in individuals with mild cognitive impairment (MCI) compared to CN participants within the amyloid‐positive spectrum,^39^ which is consistent with our finding that the A+T+ group, characterized MCI, also exhibited elevated switching rates. Interestingly, the same study reported a decline in network switching rates within the dementia group,^39^ which is consistent with our observation of a general decrease in switching rates between Braak stages V and VI across most networks. This pattern likely reflects a late‐stage breakdown in dynamic network flexibility. These findings underscore the non‐linear trajectory of dynamic network changes across the AD continuum. One potential explanation is that Aβ‐associated neuronal hyperexcitability and hyperconnectivity may transiently destabilize large‐scale network dynamics in earlier Braak stages,^40^, ^41^ resulting in increased network switching. As pathology advances, heightened activity could further increase tau vulnerability and propagation via activity‐dependent tau release,^42^ whereas progressive synaptic loss and network disconnection may constrain network reconfiguration and ultimately reduce switching. Another potential reason for inconsistent findings is the selection of time window size in dynamic FC analyses. Shorter windows may better capture transient neural dynamics but are more susceptible to noise, whereas longer windows may smooth over meaningful fluctuations. These methodological choices can significantly influence estimates of network flexibility and stability, leading to divergent interpretations across studies.^43^

Importantly, our findings support previous findings in which an elevated network switching rate is significantly associated with amyloid burden in the frontal, temporal, parietal, and occipital cortices. This spatial distribution aligns with previous longitudinal studies that identified early amyloid accumulation in these same regions during the initial stages of AD progression.^44^ In our analysis, DAN and SMN emerged as particularly vulnerable to AD‐related changes, characterized by increased network switching rates and a positive correlation with amyloid accumulation. Previous studies indicate that distal Aβ accumulation affects not only local brain structure but also impacts regional metabolism and function through long‐range functional network connectivity, particularly involving hub regions such as the DMN.^39^, ^45^, ^46^ These findings support the notion that amyloid‐induced disruptions extend beyond local effects to influence broader network‐level dynamics. A proposed mechanism underlying this effect is Aβ‐mediated hyperactivation, which is associated with impaired synaptic transmission in highly active neurons.^47^ In particular, the most active neurons appear to be at the most significant risk of dysfunction and hyperactivation,^48^ potentially contributing to widespread FC disturbances in AD.

Our study confirmed that an elevated network switching rate was significantly correlated with regional tau burden, particularly in the medial temporal and temporoparietal lobes, as well as with advancing Braak stages. This dynamic functional network pattern closely aligns with the spatial pattern of tau accumulation in patients with AD. Previous research has indicated that the increased FC in both the medial temporal lobe and the posteromedial hubs of the DMN is associated with abnormal accumulation of tau burden in the medial temporal regions.^49^ Furthermore, findings from combined fMRI and longitudinal tau PET studies suggest that Aβ‐related increases in FC within tau epicenters to typical tau‐vulnerable brain regions facilitate the annual progression of tau deposition.^12^, ^44^, ^50^ These results underscore the critical role of network connectivity changes in driving the spatial distribution of tau pathology throughout the AD continuum. In line with this, recent rs‐fMRI studies have demonstrated that in Aβ‐positive individuals with AD dementia, the relationship between tau burden and FC is not restricted to the DMN but also involves other large‐scale networks, including the FPN, DAN, and VN.^12^ Collectively, these findings support the hypothesis that Aβ and tau pathologies differentially affect multiple resting‐state networks in AD, contributing to widespread network dysfunction.

Moreover, we observed a significant interaction between amyloid burden and network switching rates in tau‐vulnerable regions, particularly in the inferior temporal neocortex corresponding to Braak stage III. This interaction was most prominent within the FPN, DAN, and SMN, suggesting that temporal instability in large‐scale functional networks may amplify the impact of amyloid pathology. Prior studies have suggested that amyloid‐related neuronal hyperactivity may stimulate the release of pathological tau, which can then be taken up by functionally connected neurons, inducing template‐based misfolding and aggregation of tau proteins.^51^ In parallel, microglial activation has been shown to directly disrupt neuronal circuits, exacerbate network instability, and promote tau‐mediated neurodegeneration.^52^, ^53^ Notably, microglial activity preferentially localizes along functionally connected brain regions^54^ and has been identified as an independent driver of both Aβ deposition and cortical atrophy in AD.^55^ Collectively, these findings support a mechanistic framework in which aberrant network dynamics and neuroinflammatory processes synergistically interact with amyloid pathology to facilitate the topographical spread of tau. This may help explain the observed interaction between elevated dynamic FC and global Aβ burden in driving tau propagation across the AD continuum.

Notably, this interaction effect emerged start from Braak stage III regions and was absent in earlier stages (I–II), which primarily involve the hippocampus and entorhinal cortex. One possible explanation is that medial temporal tau pathology and amyloid deposition may initially develop through largely independent mechanisms.^56^ Supporting this view, PET‐based Braak staging studies have shown that early Braak stage progression (0–II) can occur in both amyloid‐positive and ‐negative individuals.^7^ In addition, studies of clinically normal older adults have reported that increased hippocampal fMRI activity is associated with greater tau accumulation in the inferior temporal neocortex, but not with Aβ burden.^57^ Furthermore, it has been shown that network dysfunction in AD often begins in the posterior DMN, even before amyloid pathology becomes detectable by PET imaging.^58^ As amyloid burden increases and reaches a pathological threshold, it may promote tau spread from the DMN to other functionally connected networks, such as the FPN, DAN, and SMN, through amyloid‐related network interactions that become more prominent during Braak stages III to V.^59^ We also observed a weaker interaction effect at Braak stage VI, possibly reflecting late‐stage breakdown of network integrity,  in which widespread synaptic loss and disconnection limit dynamic reconfiguration—consistent with reduced switching in dementia. Finally, our study also found that the switching rate, in combination with amyloid levels, could accurately predict future tau accumulation. This supports the proposed framework in which elevated switching rates, particularly in the DAN, modulate the effect of amyloid on tau propagation across Braak stages.

This study has several limitations. First, its cross‐sectional study limits our ability to infer causality or temporal dynamics, and the use of interaction analysis further constrains causal interpretation. Future longitudinal or experimental studies are necessary to confirm the directionality and causality of the observed effects. In particular, longitudinal analyses of tau pathology progression may provide greater sensitivity in capturing associations with dynamic network changes, especially during the early stages of amyloid accumulation when the pathological burden is still low. Second, methodological choices, such as the specific network parcellation schemes and the sliding window parameters used to estimate network switching rates, may influence the sensitivity and specificity of dynamic connectivity metrics. Although supplementary analyses using alternative window lengths (≈ 40 seconds and ≈ 60 seconds) yielded qualitatively consistent results, further research should seek to replicate these findings in independent cohorts using standardized dynamic connectivity pipelines to enhance reproducibility and generalizability. Third, fewer significant interaction effects were observed at Braak stage VI, which may be partially due to the smaller sample size within this subgroup, limiting statistical power and the ability to detect robust associations. Future research should prioritize longitudinal investigations focused on early tau‐affected regions, such as the hippocampus and entorhinal cortex, to better capture the temporal sequence of pathological and functional changes.

Our findings support the framework that elevated network switching rate amplifies the pathological effects of amyloid accumulation, promoting tau distribution across Braak stages in AD. These results highlight network switching rate as a potential therapeutic target and offer valuable implications for early detection and clinical monitoring in AD.

## CONFLICT OF INTEREST STATEMENT

The authors have no conflicts of interest to report related to this work. Outside the work presented in this paper, P.R.N. provides consultancy services for Roche, Cerveau Radiopharmaceuticals, Lilly, Eisai, Pfizer, and Novo Nordisk. He also serves as a clinical trials investigator for Novo Nordisk and Biogen. Author disclosures are available in the supporting information.

## CONSENT STATEMENT

All human participants or their caregivers provided informed written consent to participate in the respective studies.

## Supporting information



Supporting Information

Supporting Information
