# Supplementary material for: Elevation in network dynamics amplifies amyloid‐dependent tau pathology
Source: Alzheimers Dement. 2026 Apr 14;22(4):e71354. doi: 10.1002/alz.71354 (PMC13077447; doi:10.1002/alz.71354)
Supplement: Supplementary file 2 — Supporting Information [file ALZ-22-e71354-s001.docx]

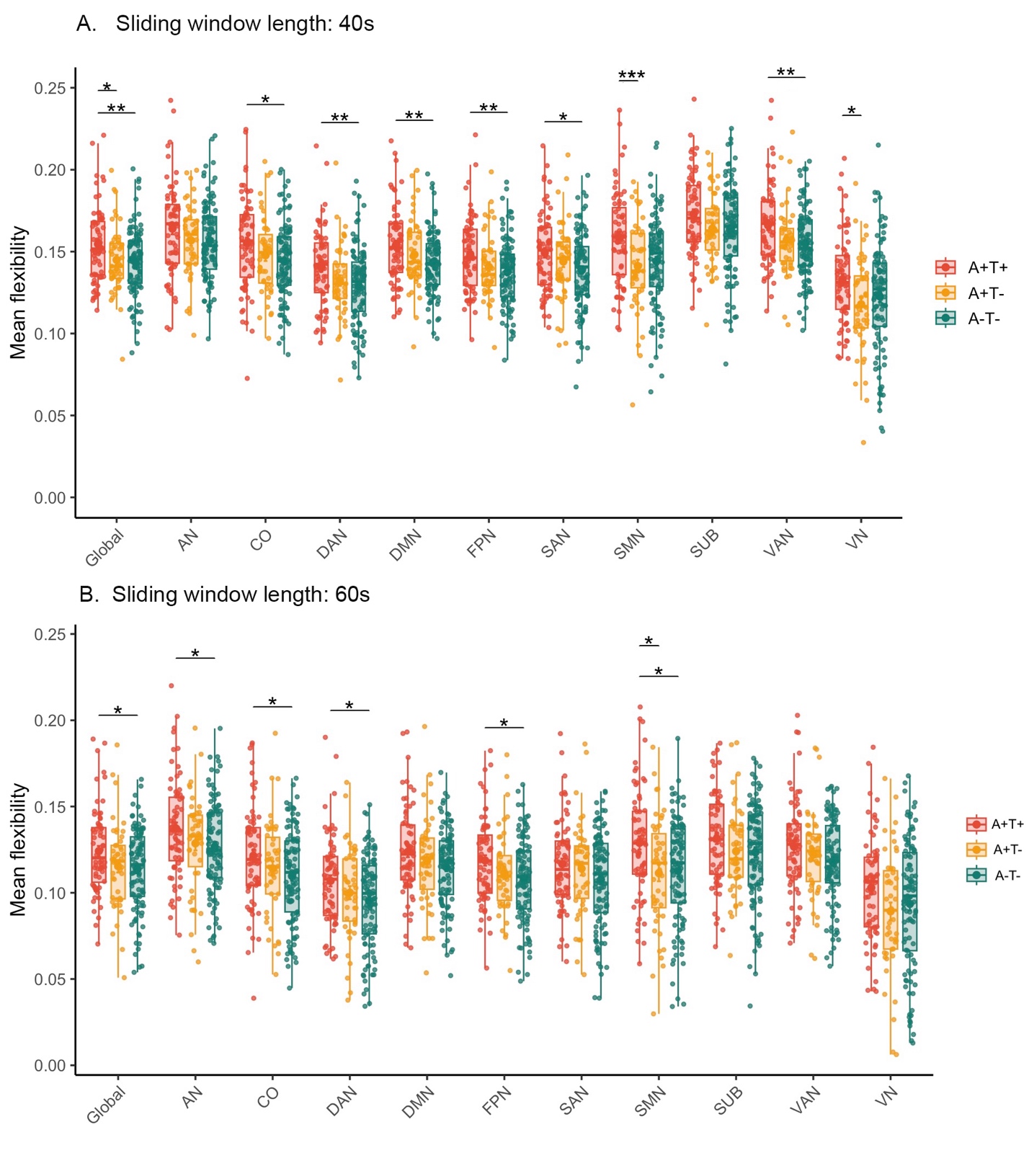


Figure S1. Network switching rate comparisons across AT groups using alternative sliding window lengths (40 s and 60 s). Group differences were assessed by one-way ANOVA with FDR correction. Error bars denote standard deviation. p < 0.05: *; p < 0.01: **.


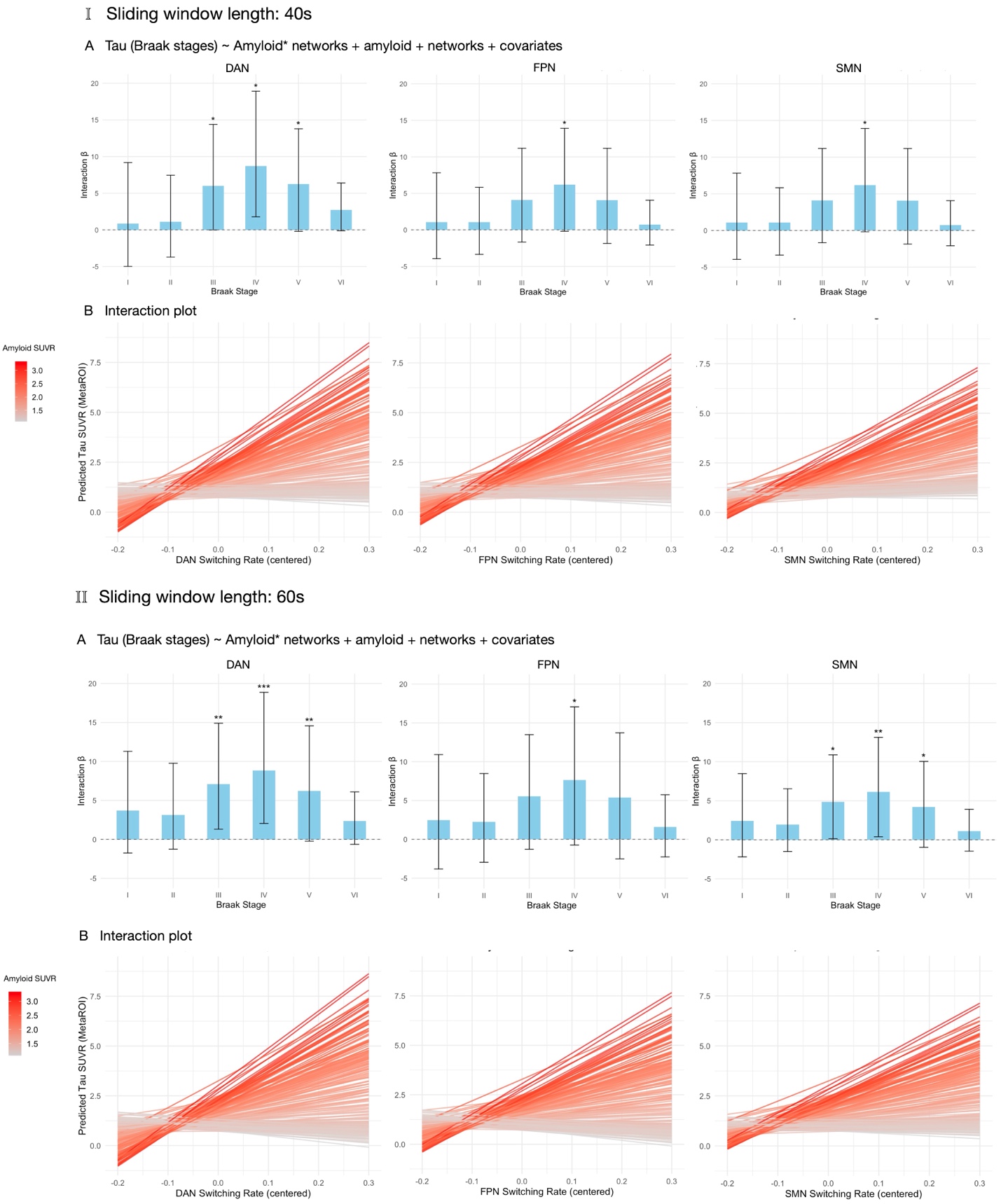


Figure S2. Interaction between network switching rate and amyloid burden on tau accumulation using alternative sliding window lengths (40 s and 60 s). Asterisks indicate statistical significance after false discovery rate (FDR) correction (p < 0.05: *; p < 0.01: **).
